# Longitudinal Changes in Brain Diffusion MRI Indices during and after Proton Beam Therapy in a Child with Pilocytic Astrocytoma: A Case Report

**DOI:** 10.3390/diagnostics12010026

**Published:** 2021-12-23

**Authors:** Lisa Novello, Nivedita Agarwal, Sabina Vennarini, Stefano Lorentini, Domenico Zacà, Anna Mussano, Ofer Pasternak, Jorge Jovicich

**Affiliations:** 1 Center for Mind/Brain Sciences (CIMeC), University of Trento, 38068 Rovereto, Italy; niveditaaga@gmail.com (N.A.); domenico.zaca@gmail.com (D.Z.); jorge.jovicich@unitn.it (J.J.); 2 Proton Therapy Center, Hospital of Trento, Azienda Provinciale per i Servizi Sanitari (APSS), 38123 Trento, Italy; sabina.vennarini@apss.tn.it (S.V.); stefano.lorentini@apss.tn.it (S.L.); 3 Radiology Unit, Santa Maria del Carmine Hospital, 38068 Rovereto, Italy; 4 Neuroradiology & Radiology Services, Scientific Institute, IRCCS “Eugenio Medea”, 23842 Bosisio Parini, Italy; 5 Pediatric Radiotherapy Service, S. Anna Hospital, A.O. Città della Salute e della Scienza, 10121 Torino, Italy; amussano@cittadellasalute.to.it; 6 Departments of Psychiatry and Radiology, Brigham and Women’s Hospital, Harvard Medical School, Boston, MA 02115, USA; ofer@bwh.harvard.edu

**Keywords:** proton therapy, free-water, diffusion MRI, astrocytoma

## Abstract

Proton beam therapy (PBT) is an effective pediatric brain tumor treatment. However, the resulting microstructural changes within and around irradiated tumors are unknown. We retrospectively applied diffusion tensor imaging (DTI) and free-water imaging (FWI) on diffusion-weighted magnetic resonance imaging (dMRI) data to monitor microstructural changes during the PBT and after 8 months in a pilocytic astrocytoma (PA) and normal-appearing white matter (NAWM). We evaluated the conventional MRI- and dMRI-derived indices from six MRI sessions (t0–t5) in a Caucasian child with a hypothalamic PA: at baseline (t0), during the PBT (t1–t4) and after 8 months (t5). The tumor voxels were classified as “solid” or “fluid” based on the FWI. While the tumor volume remained stable during the PBT, the dMRI analyses identified two different response patterns: (i) an increase in fluid content and diffusivity with anisotropy reductions in the solid voxels at t1, followed by (ii) smaller variations in fluid content but higher anisotropy in the solid voxels at t2–t4. At follow-up (t5), the tumor volume, fluid content, and diffusivity in the solid voxels increased. The NAWM showed dose-dependent microstructural changes. The use of the dMRI and FWI showed complex dynamic microstructural changes in the irradiated mass during the PBT and at follow-up, opening new avenues in our understanding of radiation-induced pathophysiologic mechanisms in tumors and the surrounding tissues.

## 1. Introduction

Proton Beam Therapy (PBT) is an effective novel approach for the treatment of a pediatric pilocytic astrocytoma (PA) [1]. However, little is known about microstructural changes that occur during the PBT [2] within irradiated tumors and the surrounding normal tissues [3]. Gaining insights into such early changes is crucial, since early responses may predict treatment outcomes [4]. Early treatment responses have been detected with diffusion-weighted magnetic resonance imaging (dMRI) in concomitant radio- and chemotherapy studies [4,5], yet have rarely been evaluated in cerebral PBT cases. Here, we investigated the microstructural changes within a PA and normal-appearing white matter (NAWM) during and after the PBT. We quantified changes in the indices derived from diffusion tensor imaging [6] (DTI) and free-water imaging (FWI) [7,8] by separately characterizing the fluid and solid PA tissue components to account for extracellular free-water (FW) accumulation.

## 2. Materials and Methods

A child (age range: 10–15 years, ethnicity: Caucasian) with a histologically determined PA underwent PBT six months after partial tumor resection. PBT was delivered in 30 daily conventional fractions of 1.8Gy relative biological effectiveness (RBE) for the total target cumulative dose of 54Gy (treatment planning details can be found in Supplementary Materials). Macroscopic tumor and target volume during treatment were monitored using structural MRI (T1-weighted or T1w, FLAIR, T2-weighted or T2w) and dMRI, which were acquired (1.5T Ingenia, Philips Medical Systems, Best, The Netherlands, see Table S1 in Supplementary Materials for acquisition parameters) before PBT (t0), during PBT (9, 17, 24, and 30 days after PBT treatment start, referred to as t1, t2, t3, and t4, and corresponding to 30%, 56%, 80%, and 100% of cumulative dose, respectively) and at 8-month follow-up (t5). The patient’s parents signed a written institutional consent to use MR data for research purposes.

**Figure 1:**
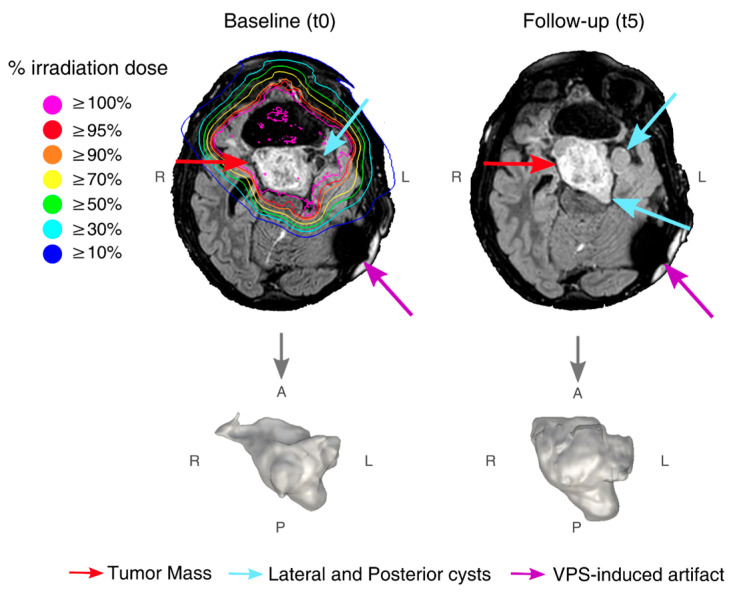
FLAIR images from t0 with overlaid radiation isodose curves, and, from t5, with the corresponding enlarged 3D render of tumor shown below. Red arrows point to tumor mass, light blue arrows point to lateral and posterior cysts (where visible), and purple arrows point to ventriculoperitoneal shunt (VPS)-induced artifact.

A ventriculoperitoneal shunt (VPS) induced an artifact (Figure 1 and Supplementary Figures S1–S3) that was excluded from all analyses. In the Supplementary Materials, dMRI preprocessing is described. DTI [6] was used to compute mean diffusivity (MD) and fractional anisotropy (FA) maps. FWI (in-house code, MatLab-R2017b, MathWorks, Natick, MA, USA) [7,8] explicitly modelled extracellular free-water and tissue compartments to compute the extracellular FW volume fraction (Figure S2) and tissue FA (FAt) maps. FAt selectively represents the FA of water molecules within or in close proximity to tissue. Longitudinal tumor volumes were computed from tumor masks manually segmented on FLAIR images by an experienced neuroradiologist (Figure 1). The image registration pipeline and NAWM segmentations are described in the Supplementary Materials.

Tumor volume was stable during treatment (see Results section), and its segmentation at t0 was used for during-treatment analyses. Tumor voxels with FW ≥ 0.95 (referred to as “fluid”) were separated from those with FW < 0.95 (referred to as “solid”). Longitudinal changes of diffusion indices in tumor and NAWM regions were evaluated using Wilcoxon rank-sum tests in R.

## 3. Results

The PBT was well-tolerated. At t5, the patient presented with vomiting, headache, and ideomotor slowing, which was effectively treated with corticosteroids.

### 3.1. Findings at Conventional MRI

**Figure 2:**
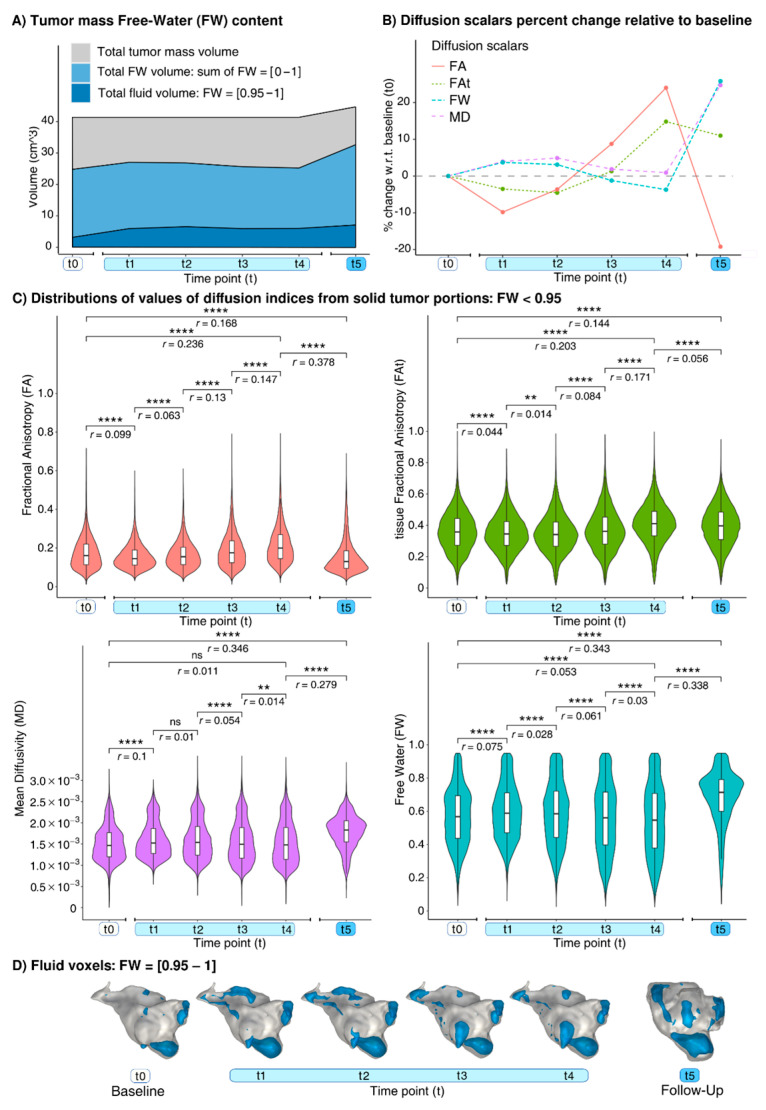
Longitudinal effects from baseline (t0) and at t1 = 9, t2 = 17, t3 = 24, and t4 = 30 days after PBT treatment start. (**A**) Volumes of tumor mass (grey), overall FW (light blue), fluid voxels (dark blue). (**B**) Percent changes with respect to t0 of tumor solid voxels median values and (**C**) their distributions. (**D**) Mapping of fluid voxels (blue) on tumor 3D renders (grey). Note: ns: not significant, **: *p* < 0.01, ****: *p* < 0.0001. The *p*-values were Bonferroni corrected for longitudinal comparisons and the four diffusion indices.

At t0, the conventional MRI (T2w and FLAIR) identified a hyperintense, partially resected mass of 41.3 cm^3^ centered in the ventral hypothalamus surrounded by two cysts on the left lateral and posterior mass aspects (Figure 1 and Supplementary Figure S1). Even though during treatment the mass showed a T2w signal increase, its volume remained stable (compared to the baseline: mean Dice coefficient of overlap: 0.95; mean relative volume change: 5.35%). At t5, the T2w signal remained high, and the tumor volume increased to 44.7 cm^3^ (+8.1%, Figure 2A).

### 3.2. Findings at dMRI

Before the PBT, the tumor presented with 7.6% fluid voxels (Figure 2A), which at t1 increased by 88.5% (Figure 2A,D). In the solid voxels, at t1, the FA and FAt decreased whereas the MD and FW increased (Figure 2B,C). Between t1 and t4, smaller changes occurred in the fluid voxels (range: (−9.5%, +11%), Figure 2A,D), and, in the solid voxels, the microstructural changes progressively inverted the trend observed between t0 and t1 (Figure 2B,C). At t5, compared to t4, the number of the fluid voxels increased by 18.1% (Figure 2A,D). In the solid voxels at t5, there were increases both in the MD (+23.6%) and in the FW (+30.7%). The DTI-FA decreased by 34.9%, and the FWI-FAt decreased by 3.3% (Table 1).

The NAWM showed microstructural changes at t5 that were the strongest in proximity to the tumor (Figures S4 and S5, Table S2).

## 4. Discussion

We detected novel longitudinal tissue microstructural changes, both during and post-treatment, in the proton-irradiated PA combining the conventional MRI and dMRI.

The diffusion MRI revealed two distinct time- and dose-dependent patterns of the microstructural changes during treatment. The first occurred at t1 (30% of dose), and the second occurred during the remaining treatment course. The first pattern of changes showed the increased FW and MD with the decreased FA and FAt, consistent with cell death, observed already after 4Gy irradiation in vitro [2], and, perhaps, damage to the PA’s fibrillated protoplasmic astrocytes and Rosenthal fibers [9]. The second pattern, characterized by modest variations observed at t2–t4 combined with the small anisotropy increases, may be related to combinations of cell apoptosis, astrocytic/microglial activation, PBT-induced ischemia, and cell swelling [2,10], with extracellular matrix alterations [11].

At t5, the increases in the total tumor volume, fluid volume, and diffusivity, reflect the observed signal increase in the T2w image and potentially indicate tumor pseudoprogression, observed in 34% of PBT-treated PA patients [12]. In addition, a 10-fold smaller decrease in the FAt relative to the FA was noticed at t5. This large FA decrease would suggest reduced cellularity and extracellular matrix reorganization in the long-term; however, the small FAt decrease observed is consistent with free-water contamination [7,8], and suggests rather minimal microstructural changes in the tissue at t5. Altogether, these observations may reflect mostly extracellular modifications, possibly explained by edema and the clearance of cellular debris.

Our follow-up dMRI findings agree with the data from the only other pediatric case-study to have investigated PA responses to PBT with a dMRI over a 7-year period [13]. Despite having a single 8-month follow-up, our analyses add novel information: (i) during the PBT, the cumulative dose causes different dMRI-detectable sequential hyperacute processes, and (ii) the additional FWI analysis, compared with the DTI alone, suggested a different interpretation regarding the responses of the solid tumor part at follow-up, thereby enhancing our specificity and our understanding of the pathophysiological mechanisms underlying a proton-beam-induced injury to a PA. As suggested by previous evidence (e.g., [4]), a tumor’s early responses to treatment might carry valuable predictive information on the therapeutic efficacy, before cellular modifications manifest at the volumetric level. Thus, monitoring the proton-irradiated tumor diffusion properties early on during a treatment course might contribute to the timely tailoring of treatments to individual patient responses, potentially leading ultimately to improved treatment outcomes.

In conclusion, the dMRI allows for the quantifying of subtle tissue changes in cystic tumors in response to PBT, which may serve as a diagnostic marker of a treatment’s effectiveness. We found that: (i) a cumulative PBT dose causes different sequential hyperacute processes during treatment, (ii) quantifying the fluid tumor content can be helpful as an additional strategy for monitoring and interpreting tissue reactions to irradiation. Larger studies with multi-shell dMRI acquisitions [14] may further investigate these metrics as predictors of a treatment’s outcome.

## Figures and Tables

**Table 1:** Longitudinal quantification of brain tumor microstructural changes during and after proton therapy treatment. Each diffusion index reports the median value of the distribution within the whole tumor volume (including fluid voxels), the relative change of the median with respect to baseline (∆) with its 1st (Q1) and 3rd (Q3) quartile values.

		Time Points (t)					
		Baseline	Proton Therapy Treatment				Follow-Up
		t0	t1	t2	t3	t4	t5
**dMRI acquisition from treatment start (days)**		−	9	17	24	30	269
**Cumulative dose (Gy) (percentage of total dose)**		none	16.2 (30%)	30.6 (56%)	43.2 (80%)	54 (100%)	none
**FA**	**median**	0.153	0.136	0.141	0.159	0.182	0.118
	**∆**	−	−11.1%	−7.8%	+3.9%	+19%	−22.9%
	**Q1** **Q3**	0.104 0.214	0.1 0.181	0.099 0.193	0.108 0.225	0.126 0.255	0.084 0.172
**FAt**	**median**	0.344	0.321	0.315	0.337	0.386	0.367
	**∆**	−	−6.7%	−8.4%	−2%	+12.2%	+6.7%
	**Q1** **Q3**	0.247 0.436	0.221 0.41	0.208 0.403	0.223 0.434	0.269 0.474	0.238 0.465
**MD** **(×10^−3^)**	**median**	1.506	1.618	1.656	1.615	1.607	1.913
	**∆**	−	+7.4%	+10%	+7.2%	+6.7%	+27%
	**Q1** **Q3**	1.215 1.855	1.321 2.191	1.294 2.285	1.217 2.211	1.193 2.215	1.615 2.249
**FW**	**median**	0.589	0.627	0.635	0.614	0.603	0.745
	**∆**	−	+6.5%	+7.8%	+4.2%	+2.3%	+26.5%
	**Q1** **Q3**	0.448 0.726	0.492 0.809	0.471 0.841	0.426 0.815	0.407 0.811	0.628 0.857

Abbreviations: t: time points; FA: fractional anisotropy; FAt: tissue fractional anisotropy; MD: mean diffusivity; FW: free-water; −: days from treatment start and ∆ are zero at baseline.

## Data Availability

Data are available on reasonable request, according to restrictions imposed by patient confidentiality.

## Supplementary Materials

Supplementary materials are available online at https://www.mdpi.com/article/10.3390/diagnostics12010026/s1.
