# Supplementary material for: Longitudinal Changes in Brain Diffusion MRI Indices during and after Proton Beam Therapy in a Child with Pilocytic Astrocytoma: A Case Report
Source: Diagnostics (Basel). 2021 Dec 23;12(1):26. doi: 10.3390/diagnostics12010026 (PMC8775026; doi:10.3390/diagnostics12010026)
Supplement: Supplementary file 1 [file diagnostics-12-00026-s001.zip › diagnostics-1502929-supplementary.pdf]

Longitudinal changes in brain diffusion MRI indices during and after proton beam therapy in a child with pilocytic astrocytoma: a case report

Lisa Novello, MSc,[+1] Nivedita Agarwal, MD,[+1,2,3,4] Sabina Vennarini, MD,[2]
Stefano Lorentini, MSc,[2] Domenico Zacà, PhD,[1] Anna Mussano, MD,[5]
Ofer Pasternak, PhD,[6] Jorge Jovicich, PhD [1]

[1] Center for Mind/Brain Sciences (CIMeC), University of Trento, Rovereto, Italy,
[2] Proton-Therapy Center, Azienda Provinciale per i Servizi Sanitari, Trento, Italy
[3] Radiology Unit, Santa Maria del Carmine Hospital, Rovereto, Italy
[4] Neuroradiology & Radiology Services, Scientific Institute, IRCCS "Eugenio Medea", 23842 Bosisio Parini, Italy;
[5] Pediatric Radiotherapy Service, S.Anna Hospital, A.O. Città della Salute e della Scienza, Torino, Italy
[6] Departments of Psychiatry and Radiology, Brigham and Women's Hospital, Harvard Medical School, Boston, MA, USA

[+] Lisa Novello and Nivedita Agarwal contributed equally to this paper

Corresponding author:
Lisa Novello,
Corso Bettini, 31,
38068 Rovereto, Trento, Italy
lisa.novello@unitn.it

SUPPLEMENTARY MATERIALS

Proton therapy treatment

The treatment plan was created in Raystion v6 (Raysearch, Stockholm) treatment planning system (TPS) and optimized according to a single field optimization technique using three non-coplanar fields (i.e.: Gantry 325° and couch 90°, Gantry 290° and couch 30°, Gantry 90° and couch 330°). Dose was calculated with a Monte Carlo dose engine over a dose grid having spatial resolution of 1.5mm. A 3 mm margin for the creation of the planning target volume (PTV) based on the clinical target volume (CTV) was used. The plan was designed to fulfill both dose prescription to target and constraints to organs at risk located nearby the target (article's Fig. 1). A PTV coverage of V98=97% (meaning the 98% of the prescribed dose covering the 97% of the PTV) was achieved in the nominal plan, while trying to maximize sparing of organs at risk: average dose to temporal lobe was 7.6Gy and 19.6Gy respectively for right and left lobe, brainstem maximum dose was 53.2Gy and average dose to left and right hippocampus was respectively 30Gy and 9.8Gy.

dMRI data preprocessing

dMRI data were denoised, and corrected for Gibbs ringing (https://www.mrtrix.org/), eddy-currents, head motion (https://fsl.fmrib.ox.ac.uk/), and bias field (http://stnava.github.io/ANTs/).

Image co-registrations for longitudinal alignment

A VPS-artifact mask was manually segmented to exclude the artifact region in registration processes (ANTs, Advanced Normalization Tools, https://github.com/ANTsX/ANTs). Each timepoint T1w was bias-field corrected and linearly registered to baseline (t0) T1w. A brain mask derived from a computed tomography acquired at baseline was registered to t0's T1w,

and a non-linear registration estimated between each time point b0-dMRI and T1w was applied to all diffusion scalar maps, which were resized to 1 mm-isotropic, and the resulting registered images were visually inspected. Bias-field corrected FLAIR (t0-t5) were linearly registered to the first time point T1w. This allowed us to have all longitudinal data aligned: tumor segmentations, diffusion scalar maps, T1w, and FLAIR images.

Normal-Appearing White Matter (NAWM) radiation dose contour volumes

T1w images were segmented using FSL's FAST (https://fsl.fmrib.ox.ac.uk/fsl), and the resulting NAWM segmentations were spatially divided based on radiation dose according to PBT contours. Four NAWM volumes were defined (Fig. S3) by % of cumulative dose: 0-10% (non-irradiated volume, NAWM0), 10-30% (NAWM1), 30-90% (NAWM2), 90-105% (NAWM3 - see also Fig. S5, left).

Microstructural changes of NAWM at follow-up

During treatment, we observed non-monotonic changes of diffusion scalars for all NAWM areas (Fig. S4). At t5, diffusion scalars changed significantly ($p<.0001$) relative to baseline, showing overall dose-dependent effect size values, effects increasing with tumor proximity (Fig. S5, percent changes are reported in Table S2).

Prior presentations of this work

Preliminary results from this work were presented as posters presentations at the International Society for Magnetic Resonance in Medicine (ISMRM) 2019 meeting in Montréal (Canada), at the ISMRM Italian chapter 2019 meeting in Milan (Italy), and at the Organization for Human Brain Mapping (OHBM) 2019 meeting in Rome (Italy).

Table S1: MRI protocol information. In bold, parameters changed w.r.t. baseline (t0). Abbreviations: FLAIR: Fluid Attenuated Inversion Recovery; dMRI: diffusion-weighted Magnetic Resonance Imaging; TSE: Turbo Spin Echo; TE: Echo Time; TR: Repetition Time; t1-t5 are defined in Table 1 in the article.

**Sequence**	**Time points (t)**	**TE (ms)**	**TR (ms)**	**TI (ms)**	**Resolution (mm)**
3D FLAIR	t0, t3-t5	357	4800	1660	0.98 x 0.98 x 0.6
	t1, t2	**351**	4800	1660	0.98 x 0.98 x 0.6

**Sequence**	**Time points (t)**	**TE (ms)**	**TR (ms)**	**Flip Angle**	**Resolution (mm)**
3D T1w	t0, t3, t4	4.5	25	25°	0.45 x 0.45 x 0.8
	t1, t2	**3.2**	**7**	**8°**	**0.47 x 0.47 x 1**
	t5	**3.3**	**7**	**8°**	**1 isotropic**
2D T2w TSE	t0, t5	100	6059	90°	0.34 x 0.34 x 5
	t1, t2	100	**6463**	90°	0.34 x 0.34 x 5
	t3, t4	100	**6665**	90°	0.34 x 0.34 x 5

**Sequence**	**Time points (t)**	**TE (ms)**	**TR (ms)**	**Flip Angle**	**Resolution (mm^3)**	**b-value (s/mm^2)**	**Number of b=0 volumes**	**Number of gradient directions**
dMRI	t0, t1	103	10117	90°	2 isotropic	800	1	32
	t2	**101**	**10029**	90°	2 isotropic	800	1	32
	t3	103	**11735**	90°	2 isotropic	800	1	32
	t4	103	**11001**	90°	**1.75 x 1.75 x 2**	800	1	32
	t5	103	**10556**	90°	2 isotropic	800	1	32

Figure S1: Conventional structural MRI showing tumor changes with different images (FLAIR, T1w, and T2w) for all time points. Acquisition parameters are listed in Table S1. R: Right; L: Left. Two cysts are visible on tumor left lateral and posterior aspects (light blue arrows). At baseline (t0) the lateral cyst presented CSF-like signal intensity. At t1, FLAIR revealed an increase in signal intensity within the cysts. During the following part of the treatment (t2-t4), the fluid within the lateral cyst returned to CSF-like FLAIR signal intensity. At follow-up (t5) FLAIR signal intensity within the lateral cyst returned to that observed in t1. The initial rise (t1) in the FLAIR signal within the cysts might reflect an increase in proteinaceous material released by cells lining the cysts due to radiation injury. Red arrows: tumor mass; purple arrows: ventriculoperitoneal shunt (VPS)-induced artifact.

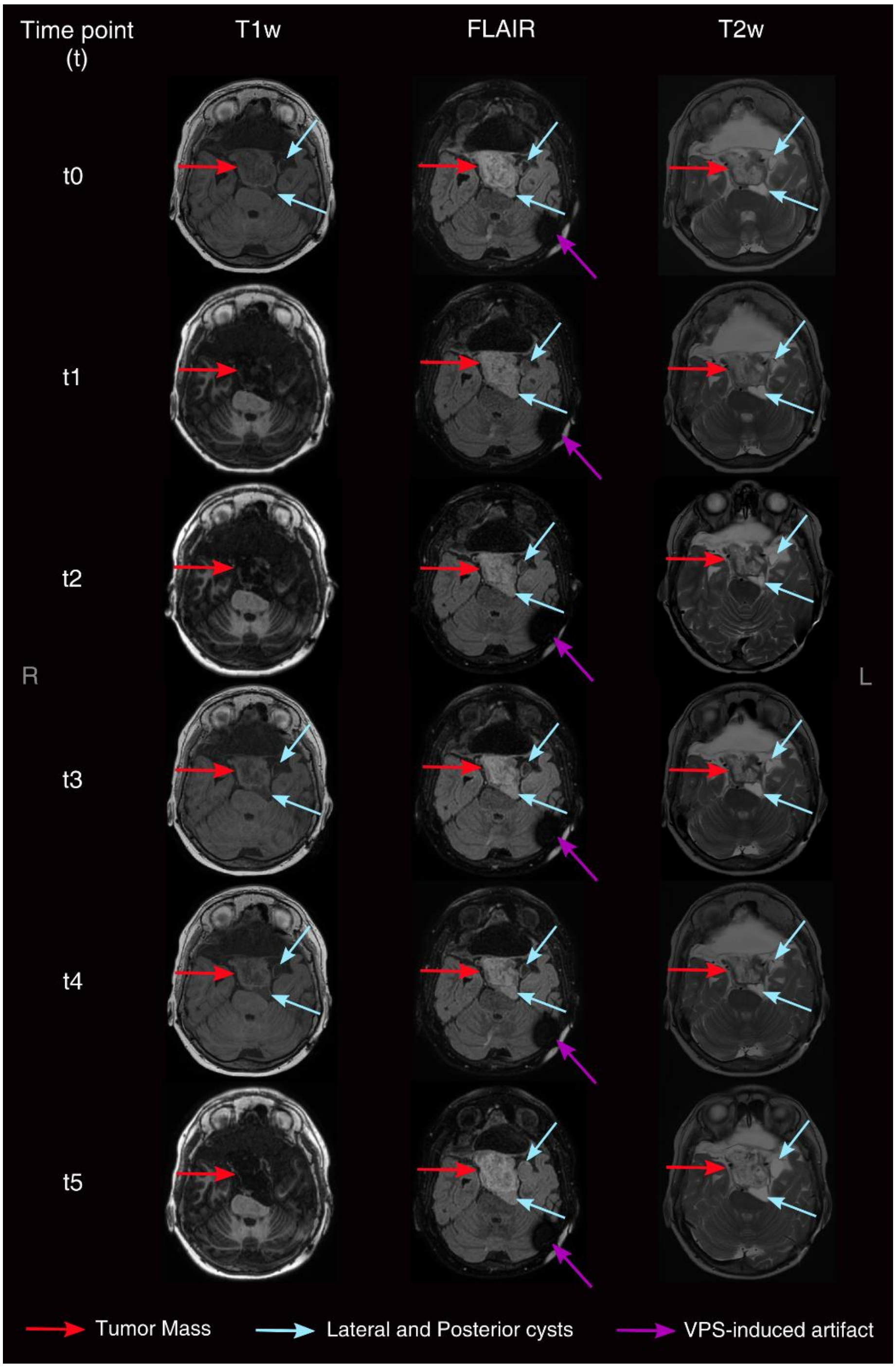

Figure S2: Diffusion scalar maps at baseline derived from the Tensor (left, Fractional Anisotropy and Mean Diffusivity) and the bi-tensor (right, Tissue Fractional Anisotropy, Free-Water) models. Red arrows: tumor mass; purple arrows: ventriculoperitoneal shunt (VPS)-induced artifact.

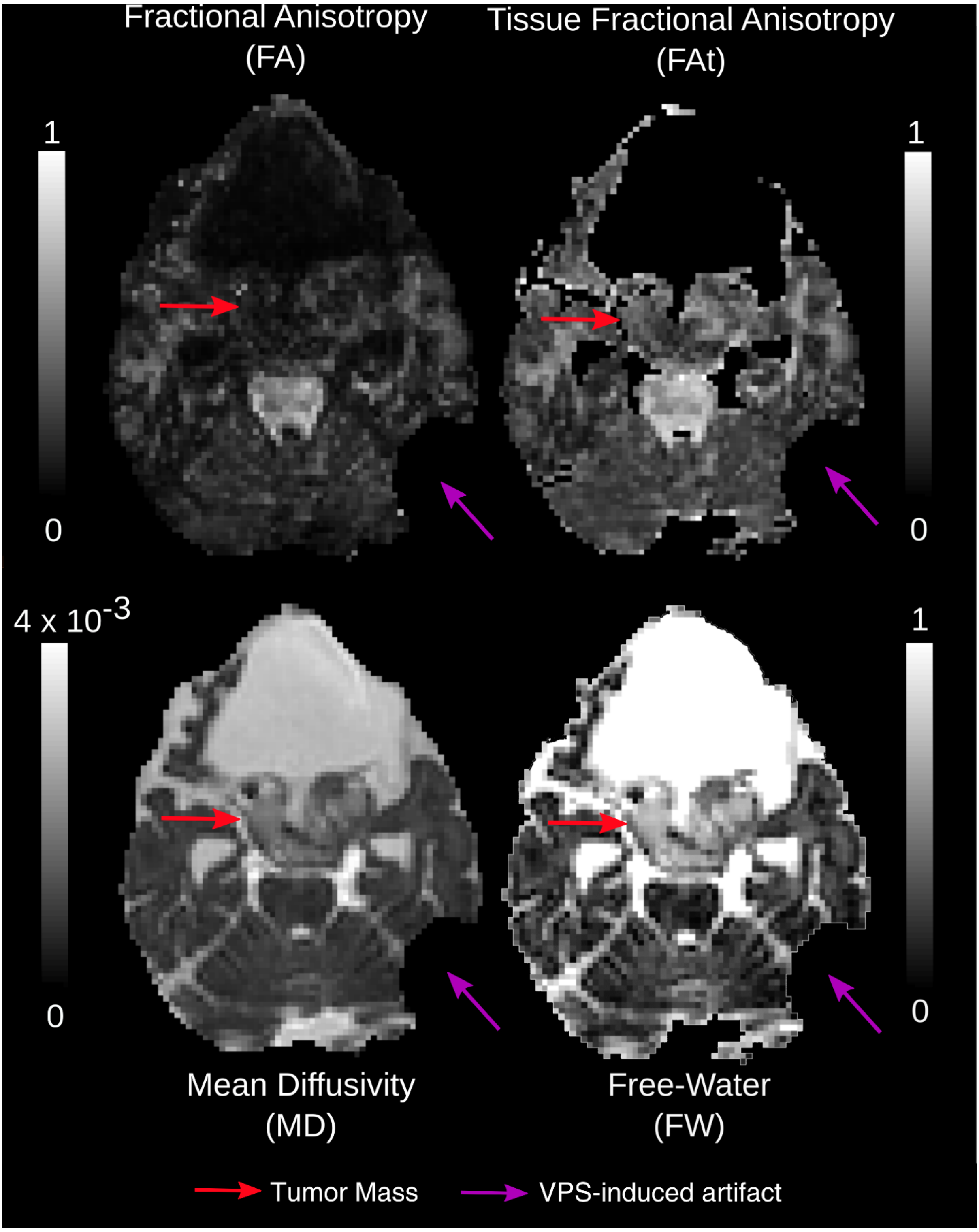

Figure S3: Mean Diffusivity (MD) map at t5 (8-month follow-up) with Normal-Appearing White Matter (NAWM) segmentations and tumor mass segmentation overlaid. NAWM regions (NAWM0, …, NAWM3) correspond to different cumulative radiation dose volumes. The procedure for their subdivision is described in the "*Normal-Appearing White Matter (NAWM) radiation dose contour volumes*" paragraph on the Supplementary Materials, and can be also seen in Figure S5.

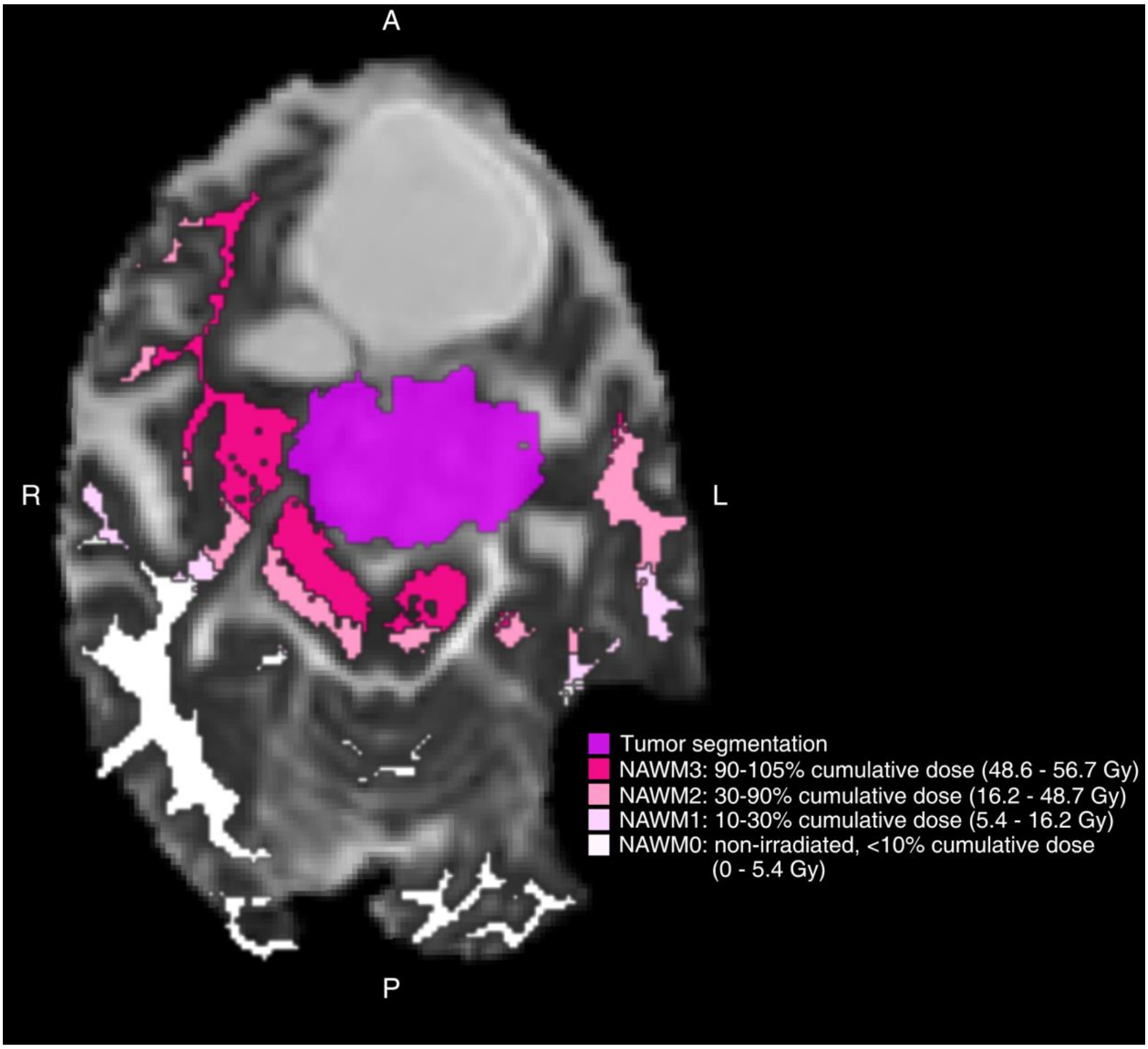

Figure S4: Relative changes (%) of Normal-Appearing White Matter (NAWM) diffusion metrics with respect to baseline (t0) for each diffusion scalar across time points (t1-t5, Table 1). NAWM regions (NAWM0, …, NAWM3) correspond to different cumulative radiation dose volumes and can be seen in Figure S5. Abbreviations: FA (Fractional Anisotropy), FAt (tissue FA), MD (Mean Diffusivity), and FW (Free-Water fraction).

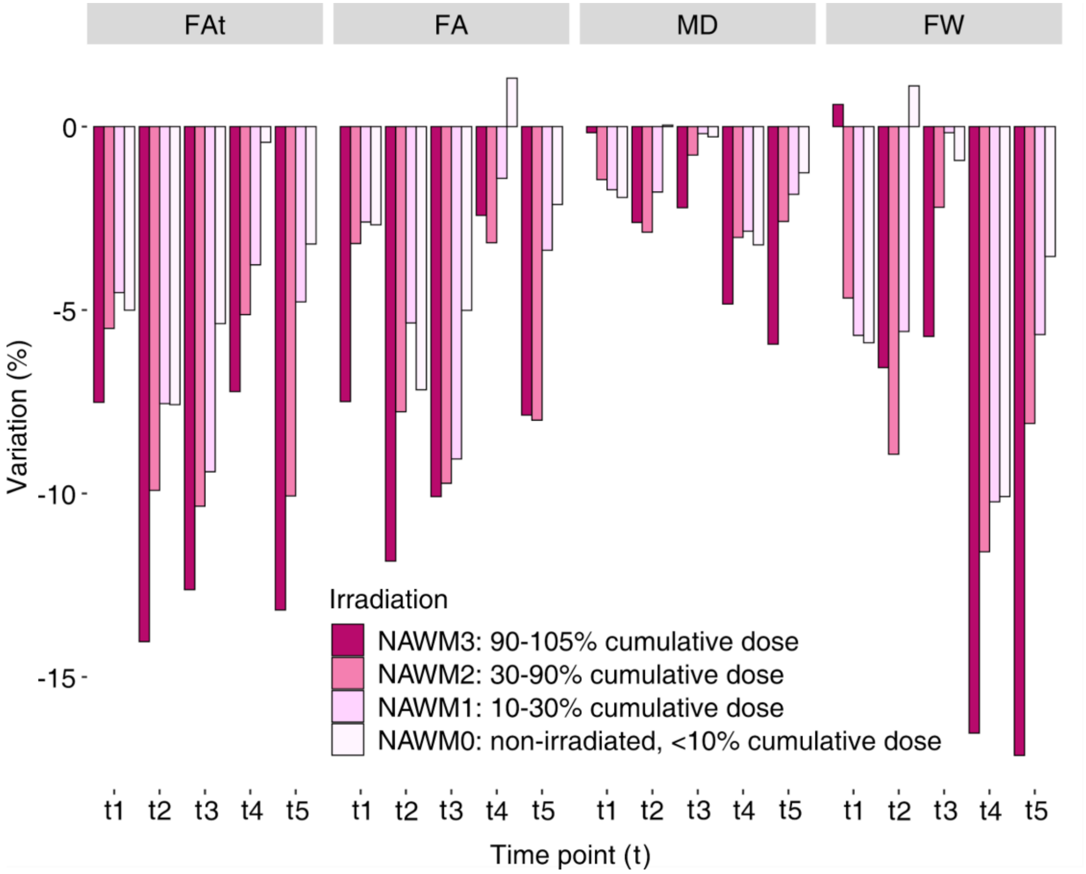

Figure S5: Left: Normal-Appearing White Matter (NAWM) segmentation color-coded for irradiation dose (in tumor-proximal to tumor-distal order): magenta: NAWM3 (cumulative dose percent range: 90 - 105%); dark pink: NAWM2 (cumulative dose percent range: 30 - 90%); light pink: NAWM1 (cumulative dose percent range: 10 - 30%); white: NAWM0 (cumulative dose percent range: <10%). Right: Wilcoxon Effect size (*r*) for t0 vs. t5 comparisons for all scalars. All comparisons revealed significant (p<.0001) differences between baseline and follow-up for each NAWM segmented region, though with overall dose-dependent effect size values. *p*-values were Bonferroni-corrected for the four-diffusion metrics and the four regions investigated. % variation for all time points can be found on Fig. S4.

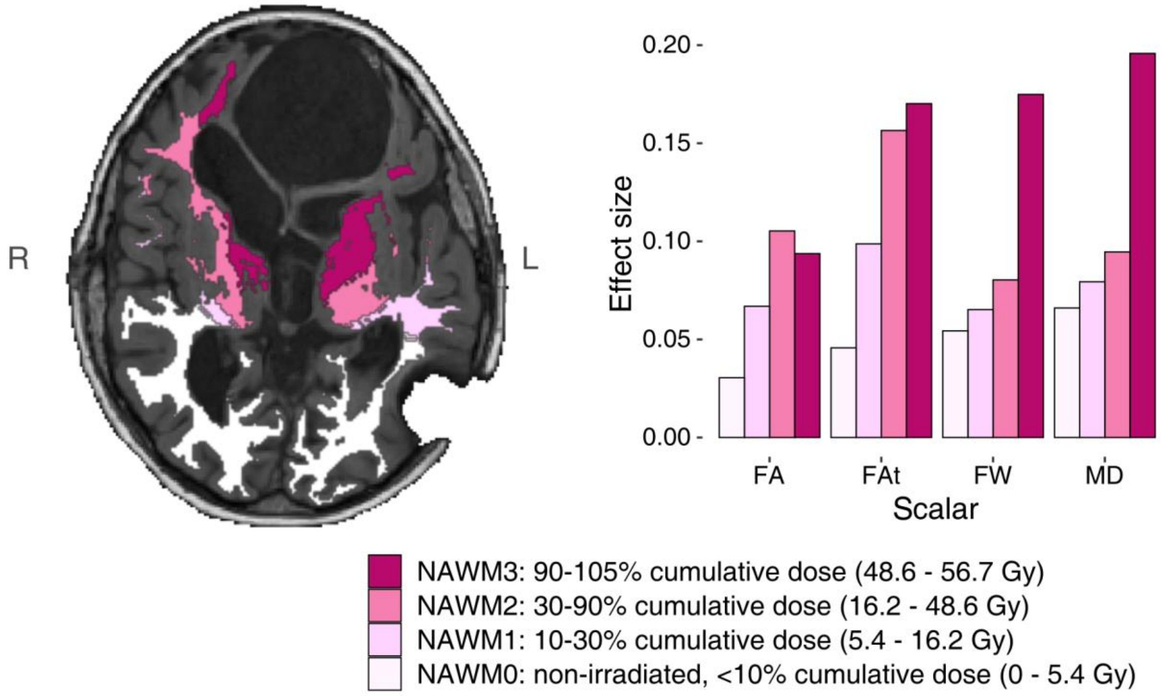

Table S2: Median Normal-Appearing White Matter (NAWM) diffusion metric values at baseline (t0) and relative changes (%) with respect to baseline for each diffusion scalar across time points (t0-t5, Table 1). NAWM regions (NAWM0, …, NAWM3) correspond to different cumulative radiation dose volumes (Figure S5). Abbreviations: dMRI (diffusion MRI), FAt (tissue Fractional Anisotropy), FA (Fractional Anisotropy), MD (Mean Diffusivity), FW (Free-Water fraction).

**dMRI metrics**	**NAWM regions**	**TIME POINTS (t)**					
		**Baseline**	**TREATMENT [1] (%)**				**FOLLOW-UP [1] (%)**
		**t0**	**t1**	**t2**	**t3**	**t4**	**t5**
**FAt**	0	0.392	-5.01	-7.58	-5.37	-0.43	-3.20
	1	0.471	-4.53	-7.55	-9.41	-3.77	-4.78
	2	0.507	-5.50	-9.92	-10.35	-5.13	-10.07
	3	0.521	-7.52	-14.04	-12.62	-7.22	-13.17
**FA**	0	0.311	-2.67	-7.17	-5.01	+1.32	-2.12
	1	0.373	-2.60	-5.35	-9.06	-1.41	-3.37
	2	0.395	-3.18	-7.77	-9.72	-3.16	-8.00
	3	0.391	-7.49	-11.84	-10.09	-2.42	-7.87
**MD**	0	$0.825 * 10^{-3}$	-1.93	+0.04	-0.28	-3.22	-1.26
	1	$0.814 * 10^{-3}$	-1.72	-1.78	-0.19	-2.85	-1.85
	2	$0.818 * 10^{-3}$	-1.44	-2.88	-0.78	-3.02	-2.59
	3	$0.839 * 10^{-3}$	-0.17	-2.61	-2.21	-4.83	-5.93
**FW**	0	0.19	-5.89	1.11	-0.92	-10.09	-3.54
	1	0.178	-5.69	-5.58	-0.17	-10.23	-5.67
	2	0.179	-4.67	-8.93	-2.20	-11.59	-8.09
	3	0.192	0.60	-6.57	-5.72	-16.53	-17.14

1: Percent changes relative to baseline
